# Temperature-Dependent Model of Rutting Behavior for Connected Layer Mixtures in Flexible Base Asphalt Pavement

**DOI:** 10.3390/ma18040808

**Published:** 2025-02-12

**Authors:** Kangning Shang, Chenguang Wan, Mengyu Guo, Chuanrong Zhou, Yingjun Jiang, Jiaolong Ren

**Affiliations:** 1 Henan Zhonggong Design & Research Group Co., Ltd., Zhengzhou 451450, China; 2 School of Highway, Chang’an University, Xi’an 710064, China; 3 School of Civil Engineering and Geomatics, Shandong University of Technology, Zibo 255049, China

**Keywords:** asphalt pavement, bonding layer, rutting behavior, type of material, temperature-dependent model

## Abstract

The flexible base asphalt pavement is characterized by its excellent performance. A critical component of this structure is the connected layer, which links the surface layer to the flexible base. This layer is located in an area of high-pressure stress distribution within the pavement. The risk of rutting is heightened in the connected layer due to the effects of loads and temperature fluctuations. However, existing studies have not established a correlation between temperature and rutting prediction models for connected layer materials. Therefore, this study aims to accurately characterize the temperature dependence of the rutting behavior of these materials. Firstly, variations in dynamic stability and rutting deformation of connected layer mixtures were investigated under different temperatures and asphalt binder types. Subsequently, a temperature-dependent model of rutting behavior was developed, highlighting the influence of varying temperatures and asphalt binder types. The proposed temperature-dependent rutting model addresses the limitations of traditional models, which fail to accurately describe the rutting behavior of materials under complex working conditions, and provides a scientific basis for optimizing asphalt pavement design and enhancing rutting performance.

## 1. Introduction

Asphalt pavement is extensively utilized in high-grade roads in China because of its advantages, including a smooth surface, absence of joints, short construction periods, and ease of maintenance and repair [1]. It plays a crucial role in modern transportation infrastructure; however, rutting has consistently been a significant issue that impacts both its service performance and lifespan [2,3]. Existing studies have demonstrated that the adhesion state at the interface between asphalt layers in flexible pavements significantly influences overall pavement performance. Appropriate interlayer bonding conditions facilitate the formation of a cohesive structure, thereby enhancing the rutting resistance of asphalt pavements. This is achieved by ensuring the integrity and bonding between layers, which enables them to withstand the stresses imposed by traffic and environmental factors. When interlayer bonding issues occur, they can lead to slippage and a reduction in shear strength between the road layers. This results in pronounced rutting problems, which not only cause a significant decline in road smoothness but also shorten the pavement’s expected service life [4,5].

The adhesive layer, a critical component of flexible pavement, behaves as a typical viscoelastic–plastic material, exhibiting significant sensitivity to variations in temperature and load [6,7,8]. Currently, the design specifications for asphalt pavement in China often rely on empirical or representative values at a specific temperature to evaluate the actual performance of the pavement. This approach does not adequately account for the variability of temperature effects and the interaction between load and environmental factors, thereby failing to accurately represent the temperature dependence of the service behavior of adhesive layer materials. In light of this, a comprehensive analysis of bonding layer characteristics, an exploration of various factors influencing rut deformation, and the development of a predictive model closely related to bonding layer temperature are crucial for scientific and accurate prediction and evaluation of rutting behavior. This approach not only provides a fundamental basis for optimizing pavement design, construction, and maintenance strategies but also ensures the long-term stability of flexible pavements and enhances road traffic safety.

The characteristics of the bonding layer are closely related to the types of materials used, and different material types can significantly affect the stress distribution in the interface area. Regarding grading types, Li et al. [9], based on fractal theory, established a connection between the rutting resistance of asphalt mixtures and fractal dimension by demonstrating a strong linear correlation between voidage and fractal dimension. Deng et al. [10] noted that the dynamic stability of mixtures at different levels exhibits minimal differences overall. According to the simulation test results by Xiao et al. [11], creep deformation is significantly influenced by materials with larger particle sizes; however, the deformation trends across different sizes are generally consistent. Collectively, these studies indicate that the rutting development patterns of mixtures are similar across various gradation types. From the perspective of asphalt type, Tong’s [12] road performance tests indicate that 30# asphalt mixture exhibits superior high-temperature rutting resistance and water stability compared to 70# asphalt mixture. Song et al. [13] analyzed the properties and high-temperature rheological characteristics of 30#, 50#, and 70# asphalt under thermal oxidation aging and ultraviolet aging. The results demonstrated that low-grade asphalt possesses enhanced anti-aging properties and improved high-temperature rheological performance during aging. Enieb et al. [14] conducted a comparative analysis of high-strength asphalt, SBS asphalt, stable rubber asphalt, and high-viscosity asphalt mixtures. Their findings revealed that high-viscosity asphalt mixtures offer exceptional high-temperature resistance and water damage resistance. Zeng and Song et al. [15,16] investigated the road performance of high-modulus natural asphalt, 50#, SBS, 90#, and 70# mixtures. The results indicated that the combination of high-modulus natural asphalt and low-grade asphalt provides excellent high-temperature rutting resistance. Collectively, these studies suggest that asphalt binders have a greater impact on rutting resistance than gradation types. In addition, pavement rutting is mainly affected by road traffic load and high temperature [17]. Wasage et al. [18] indicated that ambient temperature significantly impacts the rutting resistance of asphalt pavement. The selection and optimization of bonding layer materials can enhance performance under varying temperature conditions, particularly at elevated temperatures, thereby reducing rutting caused by heat softening [19,20]. Behnke et al. [21] noted that when considering temperature-dependent material behavior (e.g., asphalt) and interlayer bonding results, rutting depth can increase by approximately 2% by the end of the service life. Zhao et al. [22] demonstrated that high-frequency traffic loads accelerate the formation of ruts. Alae et al. [23] also observed that rutting tends to increase linearly with the number of load applications but eventually stabilizes after reaching a certain threshold of load repetitions. Therefore, establishing a temperature-dependent model of rutting behavior for connected layer mixtures is of great importance, as it can elucidate the effects of different asphalt binder types and loading conditions.

In addressing the aforementioned issues, early scholars primarily concentrated on developing predictive methods for asphalt pavement rutting, aiming to establish accurate models and evaluate the anticipated trends in pavement rutting. Si et al. [24] introduced a calculation approach for determining the equivalent temperature of asphalt pavement rutting, taking into account effective temperature and load frequency. Behnke [25] investigated the impact of temperature fluctuations in seasonal freezing zones on the permanent deformation of pavements, proposing a rutting prediction model along with the equivalent temperature values for asphalt pavement in these regions. Kim et al. [26] examined the interlayer behavior of double-layer asphalt samples collected from actual highways, employing coaxial shear tests and parallel direct shear tests to analyze how temperature variations affect the performance of the bonding layer. To address rut damage, numerous theoretical models have been proposed to predict the rut depth of various pavement structures using parameters obtained from laboratory tests, such as the Hamburg wheel test [27] and accelerated pavement tests [28,29]. Jarazi et al. [5] investigated the influence of temperature variations on the interlayer bonding state through numerical simulations and experimental tests. Their temperature-dependent model accurately describes the behavior of asphalt materials under varying temperature conditions, providing a scientific foundation for rutting prediction and pavement design [30]. Zhang et al. [31] indicate that stability under heavy load conditions is a significant concern. Through the asphalt pavement analyzer test and the China wheel load test, a relationship model was established between dynamic stability and the temperature, load, and viscosity of the binder. Traditional rutting prediction models are typically based on empirical equations or simplistic mechanical models, which fail to accurately represent material behavior under complex working conditions. Furthermore, most studies have been conducted at specific temperatures or within narrow temperature ranges, neglecting the temperature dependence of service behavior in conjunction with the temperature domain of the interlayer. Additionally, a model function linking service behavior and the temperature of the interlayer mixture has yet to be established. Therefore, a more accurate model is required to predict rutting deformation, considering factors such as temperature, load frequency, and material properties [32,33], in order to effectively characterize the temperature dependence of asphalt mixture service behavior.

Hence, this study aims to investigate the influence of laws of rutting behavior on asphalt pavement under different material types and structural combinations. Through a carefully designed indoor test, this study systematically studies the rutting characteristics of surface and bonding mixes under different conditions and analyzes the interrelationships among various factors. It establishes relevant models for rutting behavior and provides a scientific basis for optimizing asphalt pavement design, with the goal of improving the rutting resistance of asphalt pavement.

## 2. Materials and Methods

### 2.1. Materials

#### 2.1.1. Asphalt

The most widely used types of asphalt in pavement applications are 70# base asphalt and SBS-modified asphalt. In consideration of their use in specialized environments, this study selected four types of asphalts: 70#, 50#, and 30# base asphalts, along with SBS-modified asphalt. The technical properties of the asphalt binders are shown in Table 1. The asphalts used in the test were 70# base asphalt produced by Shandong Jingbo Petrochemical Co., Ltd. (Binzhou, China), 50# base asphalt and 30# base asphalt produced by Jiangsu Tianuo Road Material Co., Ltd. (Zhenjiang, China), and SBS-modified asphalt produced by Maoming Xinlu Building Materials Technology Co., Ltd (Maoming, China).

#### 2.1.2. Aggregate

(1) Coarse Aggregate

The coarse aggregate was basalt crushed stone produced by Shaanxi Rongxin Mining Development Co., Ltd. (Shangluo, China). The technical properties are shown in Table 2.

(2) Fine aggregate

The fine aggregate was limestone machine-made sand produced by Shaanxi Rongxin Mining Development Co., Ltd. The technical properties are shown in Table 3.

(3) Mineral powder

The mineral powder was limestone mineral powder produced by Shaanxi Rongxin Mining Development Co., Ltd. (Shangluo, China). The technical properties are shown in Table 4.

(4) Asphalt mixtures

AC-20 and AC-25 asphalt mixture composites were selected in this study. Their aggregate gradations are presented in Table 5. The volume parameters of different asphalt mixtures are listed in Table 6.

### 2.2. Methods

#### 2.2.1. Sample Preparation Method

**Figure 1:**
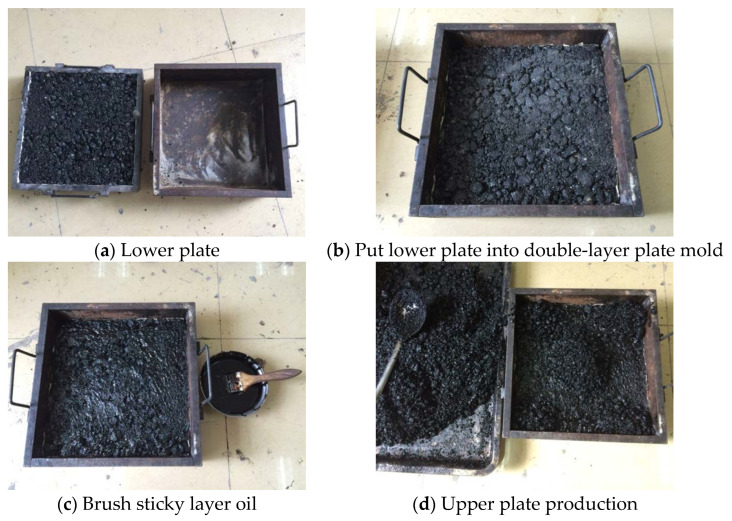
Preparation process of double-layer rutting plate.

In this study, a double-layer rutting plate sample with the size of 300 mm × 300 mm × 180 mm was used to investigate the rutting resistance of the connected layer mixtures. The sample was divided into two layers. The lower layer with the height of 120 mm was the standard flexible base mixture (i.e., ATB-30 asphalt mixtures in this study), and the upper layer with the height of 60 mm was AC-20 or AC-25 asphalt mixtures. The formation process of the double-layer rutting plate was as follows, as also illustrated in Figure 1.

(1) The Lower Plate

The quality of the asphalt mixture in the bottom layer was determined by its density, the height of the sample, and the cross-sectional area of the rut test mold. First, the thoroughly mixed asphalt layer was poured into the 12 cm high single-layer rutting plate test mold and compacted using a compacting hammer. Subsequently, a wheel roller was employed to roll the test mold containing the mixture back and forth until the thickness of the sample reached the specified height. Once this height was achieved, the wheel roller was stopped, completing the formation of the bottom layer sample.

(2) Sticky Layer Oil

After the lower laminate sample was cooled for more than 24 h, it was demolded and placed into the rut plate test mold, which measured 300 mm × 300 mm × 180 mm. The adhesive layer oil was evenly applied and allowed to set for at least 2 h.

(3) The Upper Plate

The quality of the asphalt mixture was determined by its density, the height of the sample, and the cross-sectional area of the rutting test mold. First, the fully mixed upper asphalt layer was poured into the double-layer rutting plate test mold, which contained the lower layer mixture, and was thoroughly compacted using a compaction hammer. Next, a wheel roller was employed to roll the test mold containing the mixture back and forth until the total thickness of the sample reached 18 cm. Once this thickness was achieved, the wheel roller was stopped, and the double-layer rutting plate sample was formed.

#### 2.2.2. Service Behavior of Connecting Layer—Temperature-Dependent Model

The effect of temperature and asphalt binder on the rutting behavior of the connected layer mixture was investigated, and a temperature-dependent model of rutting behavior was established.

**Figure 2:**
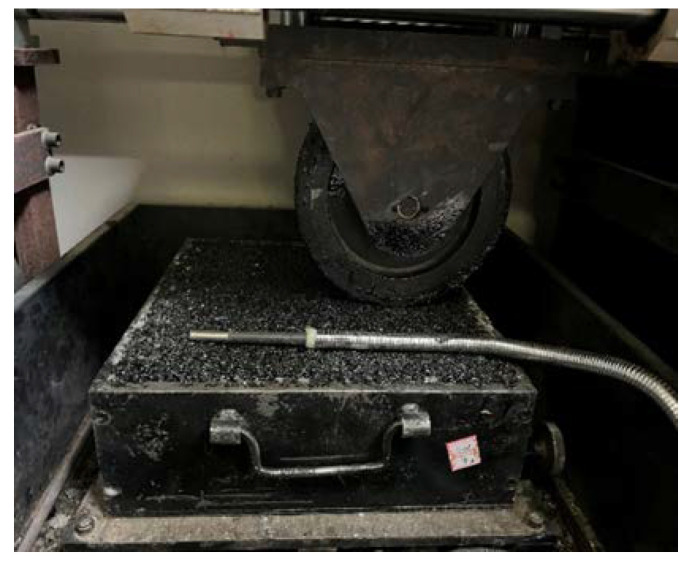
Rutting test.

(1) According to Section 2.2.1, double-layer rutting plate samples were prepared using various types of asphalt binders. This study selected four types of asphalt binders: 70#, 50#, and 30# base asphalt binders, as well as SBS-modified asphalt binder.
(2) The double-layer samples were placed in a drying oven at the target temperature for 6–8 h. To encompass a range of typical temperatures while also considering extreme conditions, this study identified six temperature levels: 10 °C, 15 °C, 20 °C, 30 °C, 45 °C, and 60 °C.
(3) According to the method T0719-2011 in “Standard Test Methods of Bitumen and Bituminous Mixtures in Highway Engineering (JTG E20-2011)” [34], an automatic asphalt mixture rutting tester was developed for the double-layer rutting plate samples (see Table 7 and Figure 2 for details of the instrument) to measure the rutting depth of these samples. The dynamic stability can be calculated using Equation (1). According to the Chinese specifications “Specifications for Design of Highway Asphalt Pavement (JTG D50-2017)” [35] and “Technical Specification for Construction of Highway Asphalt Pavements (JTG F40-2004)” [36], this study selected four types of loads: 0.5 MPa, 0.7 MPa, 0.9 MPa, and 1.1 MPa. Three samples were successfully tested.

(1) $$DS=\frac{(t_{2}-t_{1})\times N}{d_{2}-d_{1}}\times C_{1}\times C_{2}$$

where *DS* is dynamic stability in times/mm; *d*_1_ is the rut deformation at time *t*_1_ in mm, *t*_1_ is generally 45 min; *d*_2_ is the rut deformation at time *t*_2_ in mm, *t*_2_ is generally 60 min; *C*_1_ is the test machine type coefficient, which is generally 1.0; *C*_2_ is the sample coefficient, which is generally 1.0; and *N* is the round-trip rolling speed of the test wheel, which is generally 42 times/min.

(4) Through the analysis of the laws governing dynamic stability and rutting depth at various temperatures, an appropriate framework was established to elucidate the relationship between rutting behavior (dynamic stability or rutting depth) and temperature. Further details can be found in Section 3.2 and Section 4.2.2.

**Figure 3:**
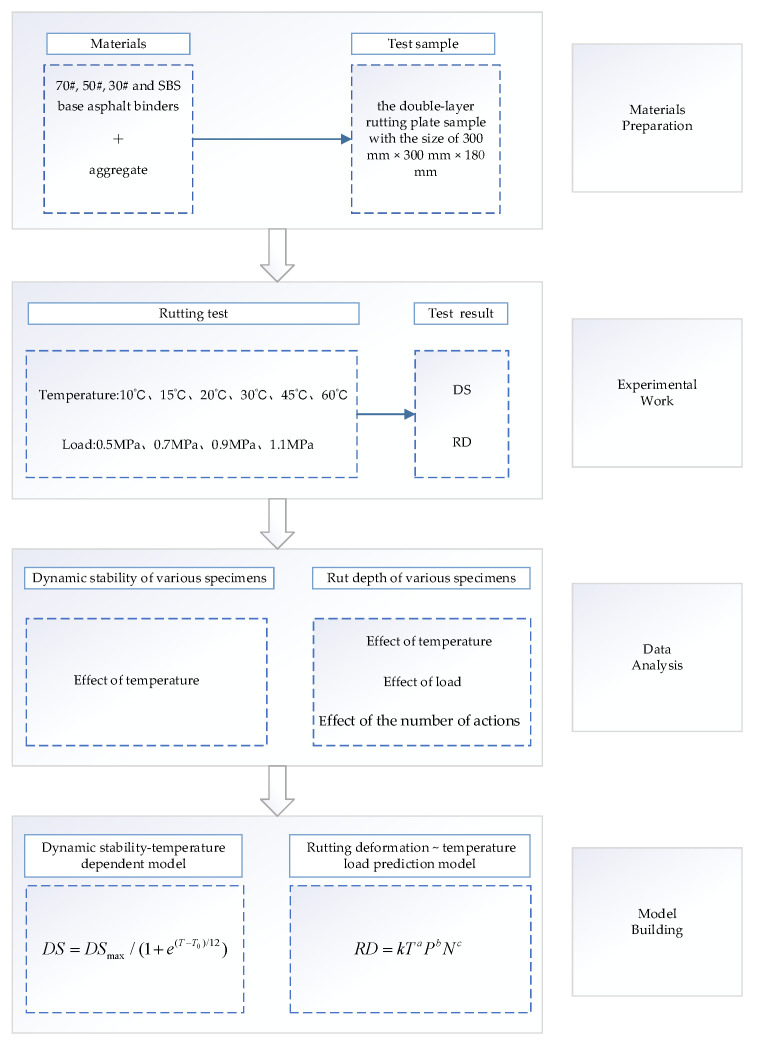
Flow chart of the rutting model establishment.

The flowchart of the above process can be found in Figure 3.

It should be noted that the impact of short-term aging of asphalt binder was not taken into account when analyzing rutting behavior. Furthermore, it was assumed that the temperature of the sample was uniformly distributed following step (2).

## 3. Dynamic Stability (DS): A Temperature-Dependent Model

### 3.1. Dynamic Stability Test Results

**Figure 4:**
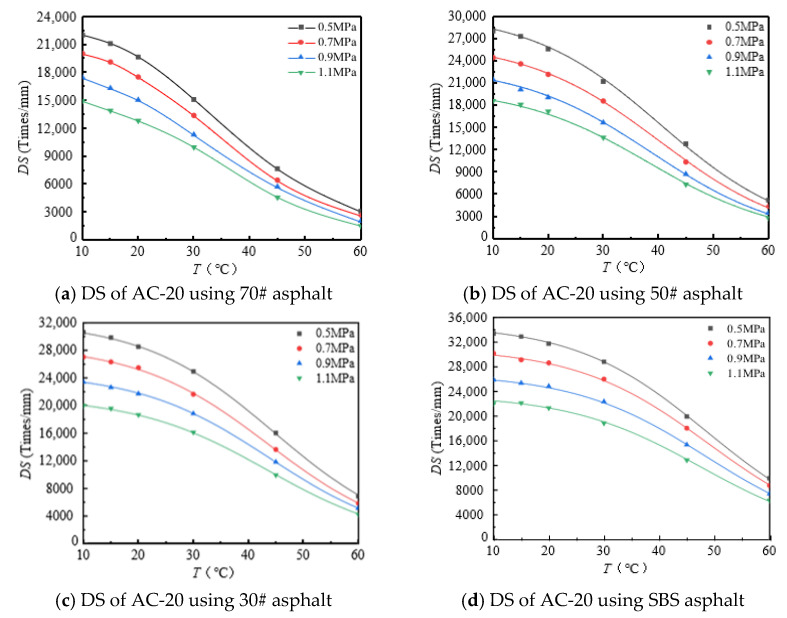
DS of AC-20 mixtures at different temperature.

**Figure 5:**
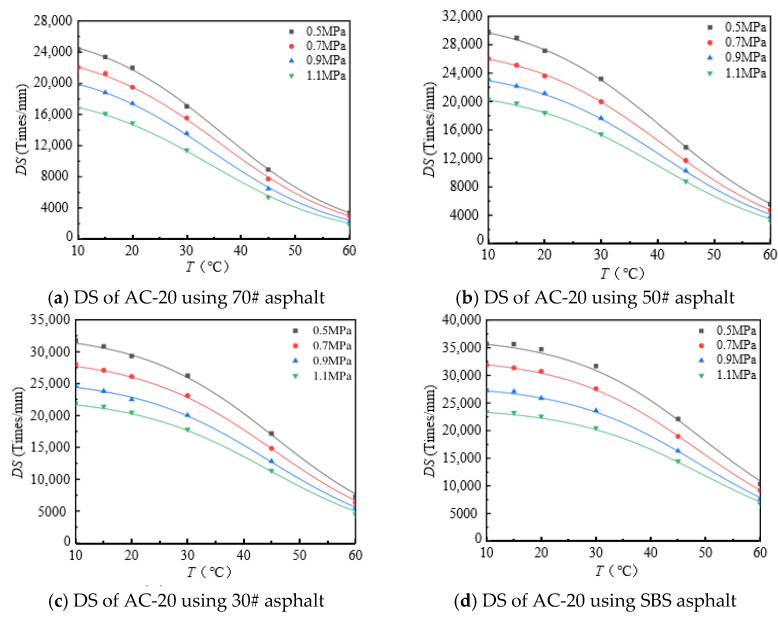
DS of AC-25 mixtures at different temperatures.

The rutting development curves for the dynamic stability test of different asphalt types and AC-20 and AC-25 mixtures are shown in Figure 4 and Figure 5.

As can be seen from Figure 4 and Figure 5, the dynamic stability of different asphalt mixtures under different loads significantly decreases with increasing temperature and tends to be stable at both high and low temperatures. The reason for this is that asphalt mixtures have strong temperature sensitivity; the higher the temperature, the lower the modulus and strength of the asphalt mixture and the weaker its resistance to deformation. At high temperatures, the mechanical properties of the mixture are mainly determined by the extruding force of the aggregate, so the dynamic stability of the mixture tends to a stable value. At low temperature, the adhesive force of asphalt determines the mechanical properties of the mixture, and the dynamic stability of the mixture tends to a stable value.

### 3.2. Dynamic Stability: A Temperature-Dependent Model Research

Based on the above analysis, a temperature-dependent model of dynamic stability was established, as shown in Equation (2). The fitting results are shown in Table 8.

(2) $$DS=DS_{\max}/(1+e^{(T-T_{0})/12})$$

where *DS*_max_ is the maximum dynamic stability in times/mm, and *T*_0_ is the target temperature in °C.

As can be seen from Table 8, the established model can represent the change rule of the dynamic stability of the mixture of the connecting layer with temperature. The correlation coefficient (*R*^2^) is around 0.99. Under the same load, the maximum dynamic stability of the mixture increases with the increase in the asphalt softening point.

## 4. Rutting Deformation (RD): A Temperature- and Load-Dependent Model

### 4.1. Results of the Rut Deformation Test

Figure 6, Figure 7, Figure 8, Figure 9, Figure 10, Figure 11, Figure 12 and Figure 13 presents the relation between rutting deformation and the loading times of different asphalt mixtures.

From Figure 6, Figure 7, Figure 8, Figure 9, Figure 10, Figure 11, Figure 12 and Figure 13, the following can be seen:

(1) With the increase in load times, rutting development curves of different asphalt types are very similar. At the beginning, the rut depth show a linear increase trend, and the increase rate of the rut depth slows down and tends to be stable in the end, which indicates that the rut shape of the connecting layer mixture will approach a limit value (RD∞) with the increase in loading times.
(2) With the increase in temperature, the rutting depth of the mixture of the connecting layer shows an increasing trend, and the rutting depth changes obviously when the temperature is greater than 30 °C.
(3) Compared to the 70# asphalt mixture, the rutting deformation of 30#, 50#, and SBS asphalt mixtures is reduced. For the AC-20 asphalt mixture, the rut depth of 30#, 50#, and SBS asphalt mixtures is reduced by 18%, 33%, and 40% on average compared to the 70# asphalt mixture under standard conditions. For AC-25 asphalt blends, 30#, 50#, and SBS asphalt blends have an average reduction in rut depth of approximately 19%, 34%, and 39% compared to 70# asphalt blends.

### 4.2. The Temperature- and Load-Dependent Model

#### 4.2.1. Influencing Factors of Rutting Deformation of Asphalt Mixture

It can be seen from Figure 5, Figure 6, Figure 7, Figure 8, Figure 9, Figure 10, Figure 11 and Figure 12 that rutting development rules of the AC-20 and AC-25 mixtures are similar under different asphalt types. Therefore, the AC-20 mixture was taken as an example when analyzing the rutting deformation factors.

(1) Effect of temperature

**Figure 14:**
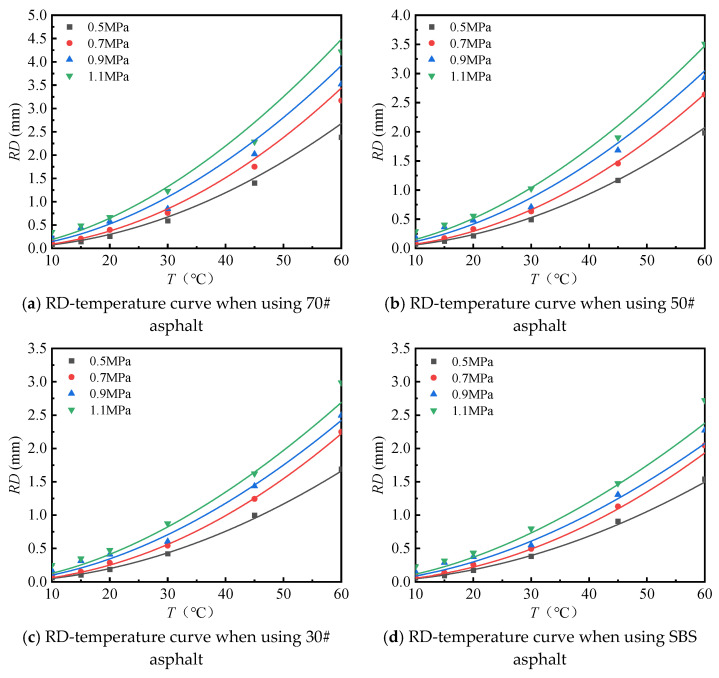
Relationship between RD and temperature when using different asphalt binders.

The rutting test results of different asphalt mixtures under different temperatures and loads were drawn, as shown in Figure 14. By fitting the test results, the functional relationship between temperature and rutting deformation under different loads was obtained, and the results are shown in Table 9.

As shown in Table 9, under different loads, the rutting deformation equation of the asphalt mixture based on temperature is a power function, and the determination coefficients (*R^2^*) are all greater than or equal to 0.94, indicating that the fitted equation can effectively predict the rutting deformation of the rutting plate.

(2) Effect of load

**Figure 15:**
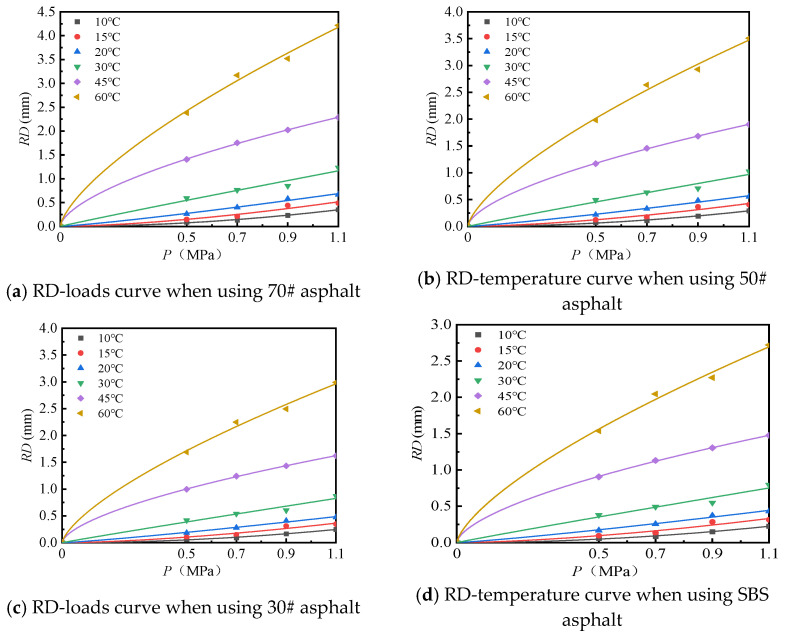
Relationship between RD and loads when using different asphalt binders.

The test results of the rutting plate under different loads and temperatures are shown in Figure 15 under 2520 loads. By fitting the test results, the relationship between load and rutting deformation at different temperatures was obtained, as shown in Table 10.

As shown in Table 10, under the action of different temperatures, the rutting deformation equations of different types of asphalt mixtures based on load are all power functions, and the determination coefficients (*R*^2^) are greater than or equal to 0.91, indicating that the fitted equation can effectively predict the rutting deformation of the rutting plate.

(3) The effect of the number of actions

**Figure 16:**
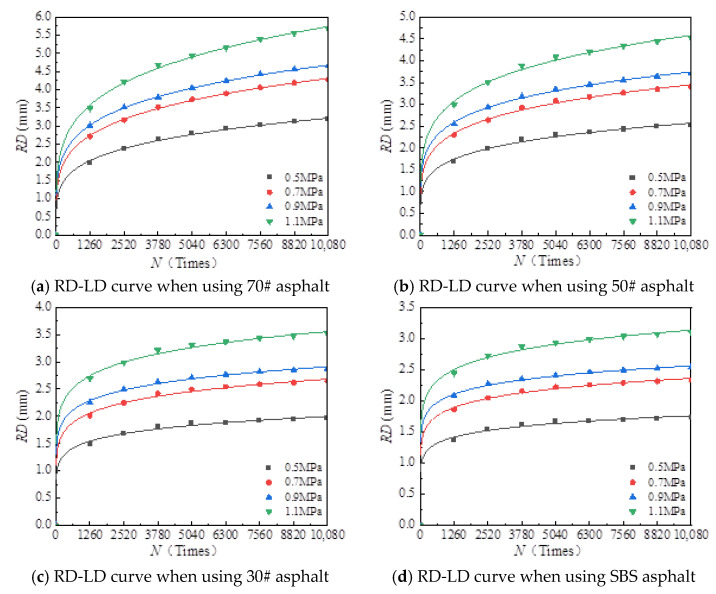
Relationship between RD and loading number (LD) when using different asphalt binders.

At the test temperature of 60 °C, rutting test results under different load times and loads were drawn, as shown in Figure 16. A fitting analysis of the test results was carried out, and the functional relationship between load times and rutting deformation under different loads was obtained, as shown in Table 11.

As shown in Table 11, under different loads, the relationship between load times and rutting deformation can still be expressed by the power function, and the determination coefficients (*R*^2^) of the fitting equation are greater than or equal to 0.91, meaning a good prediction effect.

#### 4.2.2. Rutting Estimation Model

It can be seen from the above research that the influence rules of temperature, load, and load action times on rut deformation are approximately power functions, and the rut deformation equation is shown in Equation (3):

(3) $$RD=\alpha x^{\beta }$$

where *RD* is the rutting deformation in mm, and *a* and *b* are the regression coefficients.

On this basis, the final asphalt mixture rutting prediction model is shown in Formula (4):

(4) $$RD=kT^{a}P^{b}N^{c}$$

where *T* is the test temperature in °C; *P* is the trial load in MPa; *N* is the frequency of load in times; and *k*, *a*, *b*, and c are the regression coefficients.

Parameters of the indoor rutting test model are shown in Table 12.

As can be seen from Table 12, the prediction model for rutting deformation, temperature, and load can represent the relationship between them. The determination coefficient (*R*^2^) is greater than or equal to 0.96, meaning a good prediction effect. Compared to traditional rutting prediction models [32,33,37], the proposed models operate over a wide temperature range to account for the temperature dependence of service behavior as well as the temperature conditions of the interlayer. This approach mitigates the limitations associated with empirical equations and simplistic mechanical models.

Moreover, asphalt type plays an important role in the rutting resistance of the connected layer mixtures. This variation can be attributed to the differing softening points of the four asphalts used in this study. As the softening point decreases, the bonding strength of the asphalt at elevated temperatures also increases [38], resulting in greater resistance of the mixture to high-temperature deformation. In addition, the dynamic stability of the mixture decreases with increasing temperature, while it tends to remain stable at both high and low temperatures, especially under higher loads. This behavior can be attributed to the fact that the mixture with high void content is continuously compacted during the initial loading phase. As the coarse aggregates form a skeletal structure, the asphalt mortar and fine aggregate are forced into the spaces between the skeleton, resulting in a sharp increase in rutting depth [39]. As the number of load applications increases, the void content of the mixture gradually decreases, and the sample becomes denser after repeated rolling, thereby enhancing its resistance to deformation at high temperatures. At this stage, the rut depth increases in a linear manner. Once the voids between the skeletal structure are completely filled to saturation, further increases in load applications lead to a slower increase in rut depth, which then stabilizes.

## 5. Conclusions

In this study, a temperature-dependent model of the rutting behavior of the asphalt pavement coupling layer was established. Moreover, the influence of asphalt binders and loads on rutting behavior was also analyzed. The main conclusions are as follows:

(1) The rutting behavior of the adhesive layer in asphalt pavement was thoroughly investigated. Temperature significantly affected the rutting behavior of the adhesive layer mixture. Specifically, the rutting depth increased with rising temperatures; initially, the rutting depth increased rapidly, before stabilizing under load.
(2) There were differences in rutting deformation among various asphalt types. The rutting deformation of 30#, 50#, and SBS asphalt mixtures was lower compared to that of 70# asphalt mixtures.
(3) The dynamic stability of the mixture decreased with increasing temperature, while it tended to remain stable at both high and low temperatures. As the temperature increased, the rutting depth of the mixture increased. Additionally, with an increase in the number of load applications, the rutting depth at the bonding layer initially increased rapidly before stabilizing.
(4) A correlation model was established based on the test results. The correlation between rutting deformation and the temperature–load dependence model was as high as 97%, while the correlation between dynamic stability and the temperature dependence model reached 99%.
(5) The model established by the research results provides scientific support for the design and optimization of asphalt pavement, which is highly significant. These models can accurately predict rutting deformation and dynamic stability under varying temperatures and load conditions. They provide essential data for pavement structure design, assist in the appropriate selection of asphalt types, and optimize the combination of pavement structures. This ultimately enhances the pavement’s ability to resist rutting and extends its service life.

## Figures and Tables

**Figure 6:**
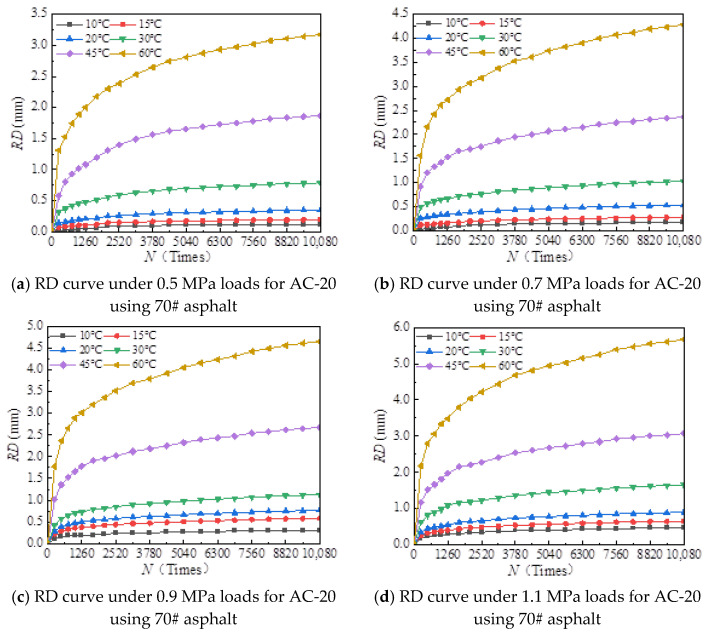
Relationship between RD and loading number (RD curve) for AC-20 mixtures using 70# asphalt.

**Figure 7:**
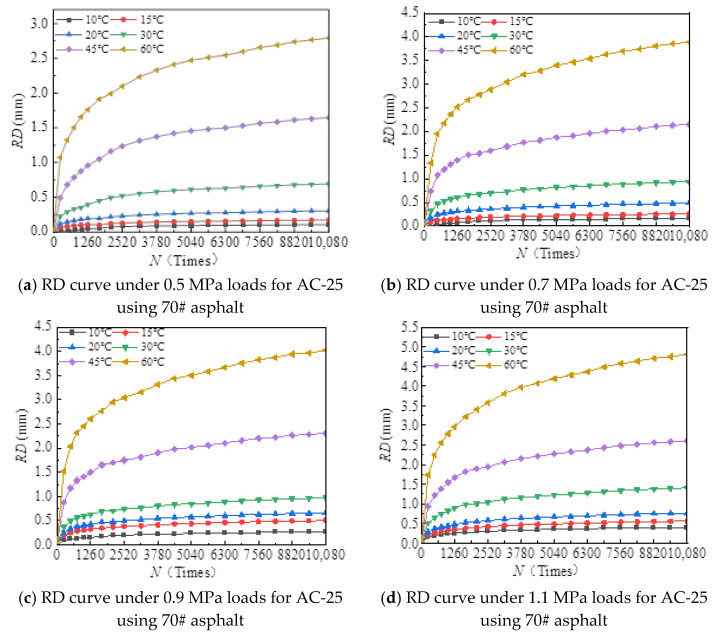
Relationship between RD and loading number (RD curve) for AC-25 mixtures using 70# asphalt.

**Figure 8:**
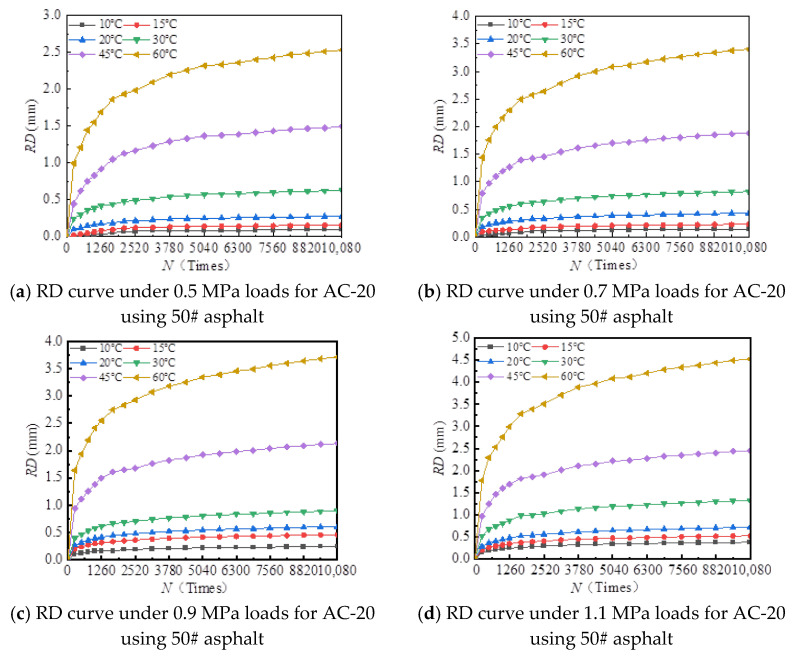
Relationship between RD and loading number (RD curve) for AC-20 mixtures using 50# asphalt.

**Figure 9:**
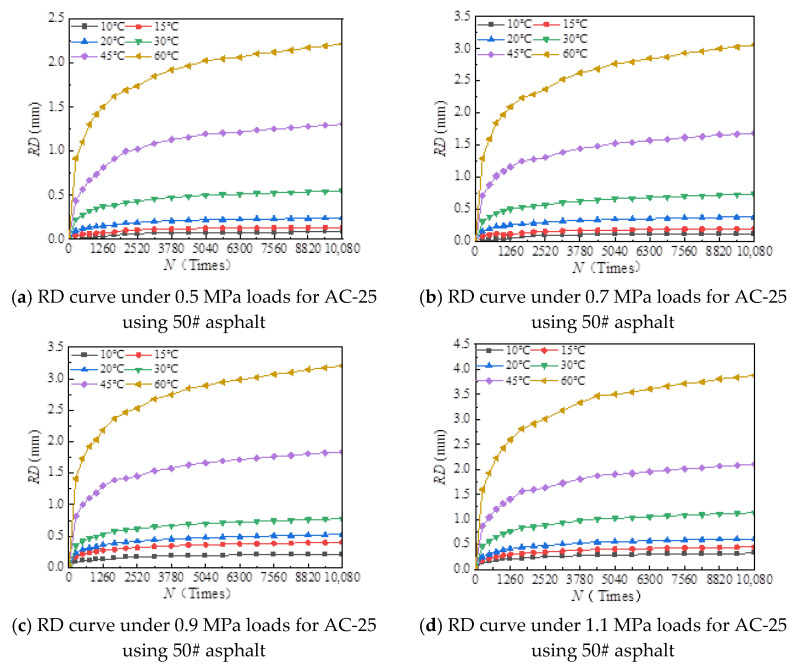
Relationship between RD and loading number (RD curve) for AC-25 mixtures using 50# asphalt.

**Figure 10:**
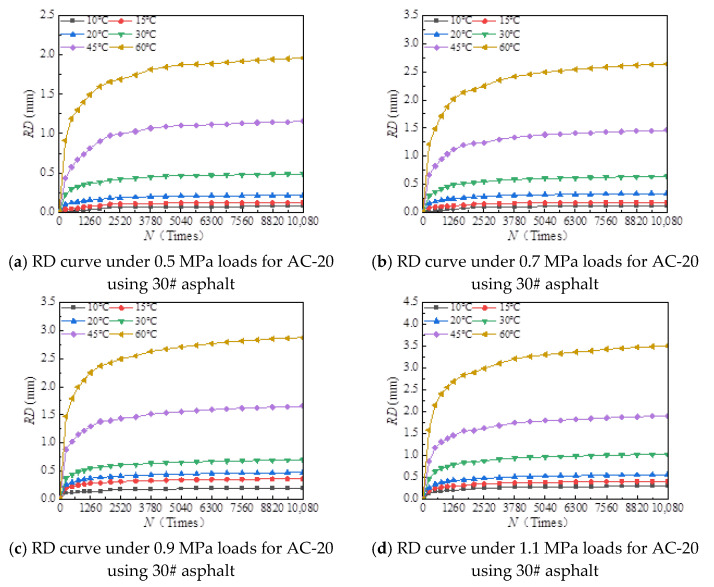
Relationship between RD and loading number (RD curve) for AC-20 mixtures using 30# asphalt.

**Figure 11:**
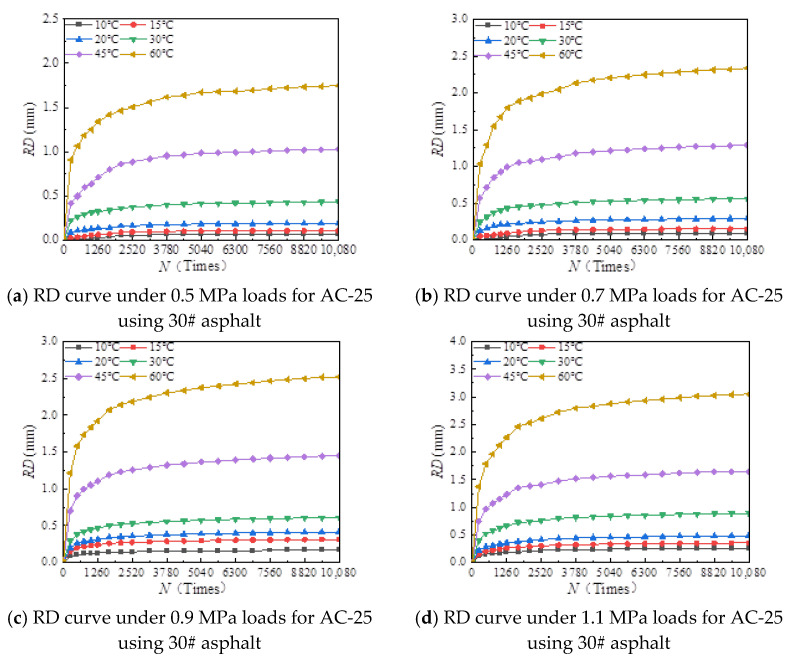
Relationship between RD and loading number (RD curve) for AC-25 mixtures using 30# asphalt.

**Figure 12:**
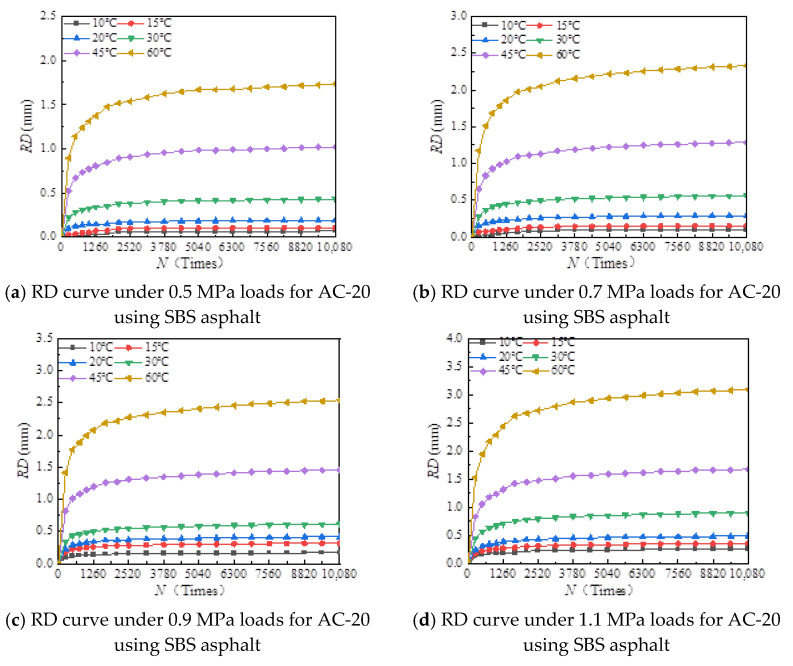
Relationship between RD and loading number (RD curve) for AC-20 mixtures using SBS asphalt.

**Figure 13:**
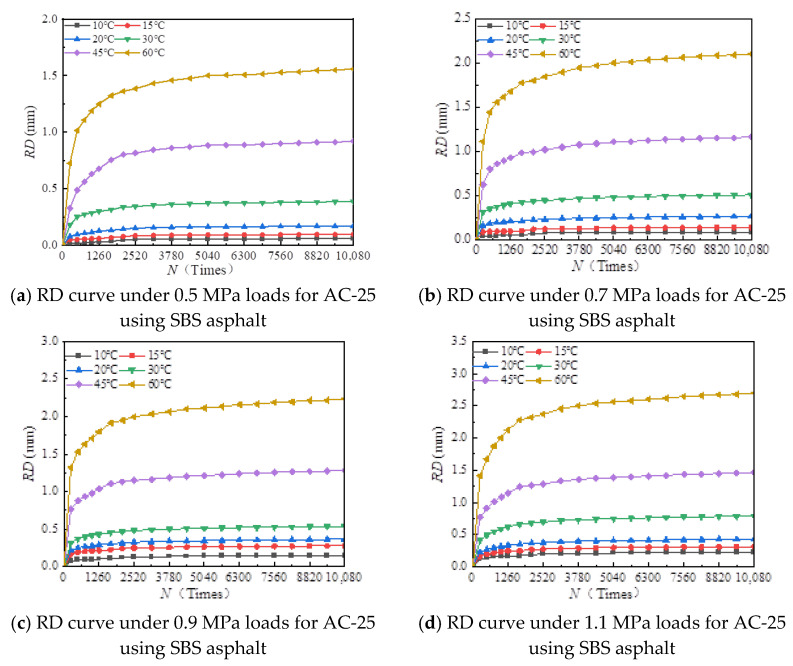
Relationship between RD and loading number (RD curve) for AC-25 mixtures using SBS asphalt.

**Table 1:** Technical properties of base asphalt.

Index		70#		50#		30#		SBS	
		Test Value	Standard Value	Test Value	Standard Value	Test Value	Standard Value	Test Value	Standard Value
Needle penetration (0.1 mm)	15 °C	22	/	17	/	13	/	16	/
	25 °C	69	60–80	52	40–60	35	20–40	47	30–60
	30 °C	98	/	71	/	52	/	58	/
PI		−0.654	−1.5–1.0	−0.385	−1.5–1.0	−0.083	−1.5–1.0	0.239	≥0
Ductility (cm)	5 °C	/	/	/	/	/	/	38	≥20
	10 °C	38	≥20	21	≥15	12	≥ 10	/	/
	15 °C	>100	≥100	84	≥80	54	≥ 50	/	/
Softening point (°C)		48	≥46	53	≥49	59	≥ 55	75	≥60
Wax content (%)		0.8	≤2.2	1.3	≤2.2	1.5	≤ 2.2	/	/
15 °C Relative density		1.018	/	1.009	/	1.021	/	1.025	/
RTFO Aging test	Quality change (%)	−0.215	≤±0.8	−0.337	≤±0.8	−0.392	≤±0.8	−0.173	≤±1.0
	25 °C Penetration ratio (%)	69	≥61	65	≥63	68	≥65	72	≥65
	5 °C Ductility (cm)	/	/	/	/	/	/	21	≥15
	10 °C Ductility (cm)	8	≥6	6	≥4	/	/	/	/

**Table 2:** Technical properties of coarse aggregates.

Index	Coarse Aggregates with the Following Size (mm)						Standard Value
	26.5–31.5	19–26.5	16–19	13.2–16	9.5–13.2	4.75–9.5	
Apparent relative density	2.862	2.857	2.837	2.825	2.820	2.818	≥2.50
Water absorption (%)	0.31	0.32	0.34	0.40	0.47	0.68	≤3.0
Needle flake particle content (%)	8.7	8.1	7.5	6.2	5.1	3.2	≤15
Crush value (%)	16.7						≤28
Los Angeles wear loss (%)	18.1						≤30
Adhesion to asphalt	5						≥4

**Table 3:** Technical properties of fine aggregates.

Index	Test Value	Standard Value
Apparent relative density	2.742	≥2.5
Robustness (parts greater than 0.3 mm) (%)	8.4	≤12
Methylene blue value (g/kg)	3.3	≤25
Angular (s)	38.7	≥30

**Table 4:** Technical properties of mineral powder.

Index		Test Value	Standard Value
Apparent relative density		2.707	≥2.5
Water content (%)		0.3	≤1
Size range (%)	<0.6 mm	100	100
	<0.15 mm	92	90–100
	<0.075 mm	78.0	75–100
Hydrophilic coefficient		0.7	<1
Plasticity index (%)		3.8	<4
Heating stability		Good	-

**Table 5:** Aggregate gradation.

Gradation	Mass Percent (%) of Aggregates Passing the Following Sieve Sizes (mm)											
	26.5	19	16	13.2	9.5	4.75	2.36	1.18	0.6	0.3	0.15	0.075
AC-25	100.0	66.4	59.6	52.9	46.2	32.7	24.5	18.6	14.4	11.1	8.3	4.6
AC-20	/	100.0	66.5	56.5	46.4	33.0	24.8	18.8	14.5	11.2	8.4	4.6

**Table 6:** Volume parameters of different asphalt mixtures.

Asphalt	Gradation	Asphalt/Aggregate Ratio (%)	Bulk Density (g/cm^3^)	Porosity (%)	VFA (%)	VMA (%)	Marshall Stability (kN)	Flow Value (0.1 mm)
70#	AC-20	4.1	2.507	4.5	65.9	13.1	12.0	31
	AC-25	3.9	2.516	4.7	65.1	13.0	12.4	30
50#	AC-20	4.2	2.509	4.2	67.8	13.1	12.8	31
	AC-25	4.0	2.518	4.5	65.7	13.0	13.1	32
30#	AC-20	4.2	2.501	4.5	66.2	13.4	13.6	31
	AC-25	4.0	2.515	4.6	65.1	13.1	14.4	30
SBS	AC-20	4.3	2.504	4.3	68.1	13.4	14.7	28
	AC-25	4.1	2.512	4.5	65.8	13.3	15.5	26

**Table 7:** Detailed specification of the rut tester.

Instrument Model	HYCZ-5C	Manufacturer	Beijing Aerospace Keyu Test Instrument Co., Ltd. (Beijing, China)
Displacement measurement range	0~50 mm ± 0.01 mm	Wheel rolling strength	0.5 ± 0.05 Mpa~1.0 ± 0.05 Mpa (by adding optional accessories, 1.4 Mpa heavy-duty traffic road rutting test can be completed)
Wheel rolling speed	42 ± 1 times/min	Temperature control	70 ± 0.5 °C (other temperatures can be used as required)

**Table 8:** Model fitting coefficient.

Load (MPa)	The Fitting Parameters	AC-20				AC-25			
		70#	50#	30#	SBS	70#	50#	30#	SBS
0.5	*DS_max_*	24,911	30,480	32,236	34,862	27,233	31,811	32,941	36,932
	*T*_0_	35.4	40.6	44.7	48.7	36.5	41.5	45.7	49.5
	*R* ^2^	0.99	0.99	0.99	0.99	0.99	0.99	0.99	0.99
0.7	*DS_max_*	22,941	26,621	28,747	31,074	24,559	27,934	29,160	33,208
	*T*_0_	34.0	39.9	43.7	48.9	36.2	41.0	45.3	48.5
	*R* ^2^	0.99	0.99	0.99	0.99	0.99	0.99	0.99	0.99
0.9	*DS_max_*	19,975	23,373	24,785	26,888	22,447	24,742	25,777	28,284
	*T*_0_	33.4	38.6	43.9	48.5	34.6	40.7	44.7	48.6
	*R* ^2^	0.99	0.99	0.99	0.99	0.99	0.99	0.99	0.99
1.1	*DS_max_*	16,578	20,386	21,249	23,471	19,135	21,849	22,881	24,160
	*T*_0_	34.2	38.4	43.5	47.6	34.4	40.2	44.5	49.5
	*R* ^2^	0.99	0.99	0.99	0.99	0.99	0.99	0.99	0.99

**Table 9:** Temperature-rut deformation function relationship.

Asphalt Type	Load (MPa)	Fit the Equation	*R* ^2^
70#	0.5	$RD=7.78\times 10^{-4}T^{1.99}$	0.97
	0.7	$RD=8.80\times 10^{-4}T^{2.02}$	0.98
	0.9	$RD=21.8\times 10^{-4}T^{1.83}$	0.95
	1.1	$RD=33.3\times 10^{-4}T^{1.76}$	0.97
50#	0.5	$RD=6.05\times 10^{-4}T^{1.97}$	0.99
	0.7	$RD=7.37\times 10^{-4}T^{2.00}$	0.97
	0.9	$RD=18.4\times 10^{-4}T^{1.81}$	0.98
	1.1	$RD=28.0\times 10^{-4}T^{1.74}$	0.98
30#	0.5	$RD=5.90\times 10^{-4}T^{1.94}$	0.99
	0.7	$RD=6.43\times 10^{-4}T^{1.99}$	0.99
	0.9	$RD=16.6\times 10^{-4}T^{1.78}$	0.99
	1.1	$RD=24.5\times 10^{-4}T^{1.71}$	0.97
SBS	0.5	$RD=5.53\times 10^{-4}T^{1.93}$	0.99
	0.7	$RD=5.82\times 10^{-4}T^{1.98}$	0.99
	0.9	$RD=14.8\times 10^{-4}T^{1.77}$	0.98
	1.1	$RD=22.6\times 10^{-4}T^{1.70}$	0.96

**Table 10:** Load-rutting deformation function relationship.

Asphalt Type	Temperature (°C)	Fitted Equation	*R* ^2^
70#	10	$RD=0.290P^{1.952}$	0.98
	15	$RD=0.443P^{1.584}$	0.91
	20	$RD=0.615P^{1.173}$	0.97
	30	$RD=1.064P^{0.959}$	0.92
	45	$RD=2.160P^{0.610}$	0.99
	60	$RD=3.911P^{0.691}$	0.97
50#	10	$RD=0.241P^{1.958}$	0.98
	15	$RD=0.368P^{1.575}$	0.92
	20	$RD=0.512P^{1.175}$	0.97
	30	$RD=0.885P^{0.958}$	0.91
	45	$RD=1.797P^{0.611}$	0.99
	60	$RD=3.254P^{0.691}$	0.97
30#	10	$RD=0.205P^{1.951}$	0.98
	15	$RD=0.314P^{1.581}$	0.92
	20	$RD=0.436P^{1.172}$	0.97
	30	$RD=0.754P^{0.961}$	0.92
	45	$RD=1.531P^{0.612}$	0.99
	60	$RD=2.774P^{0.693}$	0.97
SBS	10	$RD=0.187P^{1.965}$	0.98
	15	$RD=0.286P^{1.582}$	0.92
	20	$RD=0.397P^{1.171}$	0.97
	30	$RD=0.687P^{0.960}$	0.92
	45	$RD=1.393P^{0.612}$	0.99
	60	$RD=2.523P^{0.692}$	0.97

**Table 11:** Load times—rutting deformation equation.

Asphalt Type	Temperature (°C)	Fitted Equation	*R* ^2^
70#	0.5	$RD=0.438N^{0.216}$	0.99
	0.7	$RD=0.580N^{0.218}$	0.99
	0.9	$RD=0.678N^{0.210}$	0.99
	1.1	$RD=0.710N^{0.227}$	0.97
50#	0.5	$RD=0.465N^{0.185}$	0.98
	0.7	$RD=0.613N^{0.187}$	0.99
	0.9	$RD=0.732N^{0.177}$	0.99
	1.1	$RD=0.781N^{0.192}$	0.91
30#	0.5	$RD=0.639N^{0.124}$	0.95
	0.7	$RD=0.831N^{0.127}$	0.97
	0.9	$RD=1.010N^{0.115}$	0.98
	1.1	$RD=1.130N^{0.124}$	0.97
SBS	0.5	$RD=0.674N^{0.104}$	0.94
	0.7	$RD=0.888N^{0.106}$	0.98
	0.9	$RD=1.076N^{0.094}$	0.98
	1.1	$RD=1.152N^{0.109}$	0.97

**Table 12:** Parameters of the rutting prediction model.

Mixture Type	Asphalt Type	K	a	b	c	*R* ^2^
AC-20	SBS	0.0002	1.802	0.921	0.237	0.976
	30#	0.0002	1.829	0.974	0.243	0.974
	50#	0.0002	1.878	1.062	0.249	0.973
	70#	0.0002	1.901	1.113	0.257	0.968
AC-25	SBS	0.0003	1.696	0.838	0.235	0.975
	30#	0.0003	1.718	0.881	0.237	0.977
	50#	0.0003	1.750	0.974	0.244	0.974
	70#	0.0003	1.783	1.037	0.253	0.966

## Data Availability

The original contributions presented in this study are included in the article. Further inquiries can be directed to the corresponding authors.
